# Adverse events of bevacizumab for triple negative breast cancer and HER-2 negative metastatic breast cancer: A meta-analysis

**DOI:** 10.3389/fphar.2023.1108772

**Published:** 2023-01-30

**Authors:** Xueqiong Xun, Jun Ai, Fuhui Feng, Pan Hong, Saroj Rai, Ruikang Liu, Baowen Zhang, Yeming Zhou, Huiyong Hu

**Affiliations:** ^1^ Department of thyroid and breast Surgery, First People’s Hospital of Qujing, Qujing, China; ^2^ Department of Orthopaedic Surgery, Union Hospital, Tongji Medical College, Huazhong University of Science and Technology, Wuhan, China; ^3^ Department of Orthopedics, Al Ahalia Hospital, Abu Dhabi, United Arab Emirates; ^4^ Department of Endocrinology, Union Hospital, Tongji Medical College, Huazhong University of Science and Technology, Wuhan, China; ^5^ Basic medical school, Tongji Medical College, Huazhong University of Science and Technology, Wuhan, China

**Keywords:** triple-negative breast cancer (TNBC), HER-2 negative metastatic breast cancer (HER-2 negative MBC), bevacizumab, adverse events (AEs), meta-analysis

## Abstract

**Background:** Triple-negative breast cancer (TNBC) and HER-2 negative metastatic breast cancer (HER-2 negative MBC) are intractable to various treatment schemes. Bevacizumab as a novel anti-VEGF drug, its safety for these two high-risk breast cancers remains controversial. Therefore, we conducted this meta-analysis to assess the safety of Bevacizumab for TNBC and HER-2 negative MBC.

**Methods:** We searched Medline, Embase, Web of science and Cochrane databases updated to 1 Oct 2022 for relevant randomized controlled trials (RCTs). In all, 18 RCTs articles with 12,664 female patients were included. We used any grade Adverse Events (AEs) and grade ≥3 AEs to assess the AEs of Bevacizumab.

**Results:** Our study demonstrated that the application of Bevacizumab was associated with increased incidence of grade ≥3 AEs (RR = 1.37, 95% CI 1.30–1.45, Rate: 52.59% vs. 41.32%). Any grade AEs (RR = 1.06, 95% CI 1.04–1.08, Rate: 64.55% vs. 70.59%) did not show a significant statistical difference in both overall results and among the subgroups. In subgroup analysis, HER-2 negative MBC (RR = 1.57, 95% CI 1.41–1.75, Rate: 39.49% vs. 25.6%), dosage over 15 mg/3w (RR = 1.44, 95% CI 1.07–1.92, Rate: 28.67% vs. 19.93%) and endocrine therapy (ET) (RR = 2.32, 95% CI 1.73–3.12, Rate: 31.17% vs. 13.42%) were associated with higher risk of grade ≥3 AEs. Of all graded ≥3 AEs, proteinuria (RR = 9.22, 95%CI 4.49–18.93, Rate: 4.22% vs. 0.38%), mucosal inflammation (RR = 8.12, 95%CI 2.46–26.77, Rate: 3.49% vs. 0.43%), palmar-plantar erythrodysesthesia syndrome (RR = 6.95, 95%CI 2.47–19.57, Rate: 6.01% vs. 0.87%), increased Alanine aminotransferase (ALT) (RR = 6.95, 95%CI 1.59–30.38, Rate: 3.13% vs. 0.24%) and hypertension (RR = 4.94, 95%CI 3.84–6.35, Rate: 9.44% vs. 2.02%) had the top five risk ratios.

**Conclusion:** The addition of Bevacizumab for TNBC and HER-2 negative MBC patients showed an increased incidence of AEs especially for grade ≥3 AEs. The risk of developing different AEs varies mostly dependent on the type of breast cancer and combined therapy.

**Systematic Review Registration**: [https://www.crd.york.ac.uk/PROSPERO/#recordDetails], identifier [CRD42022354743].

## 1 Introduction

Among all subtypes of breast cancer, the prognosis of triple-negative breast cancer (TNBC) and HER-2 negative metastatic breast cancer (HER-2 negative MBC) remains intractable to various treatment regimens. TNBC, devoid of estrogen receptors (ERs), progesterone receptors (PRs) or human epidermal growth factor receptor 2 (HER-2), is highly invasive due to its high proliferative capacity and elevated vascular endothelial growth factor receptor (VEGFR) level, which can promote angiogenesis (Nalwoga et al., 2011). It is difficult to treat patients with TNBCs with endocrine therapy or other treatments targeting those three receptors (Foulkes et al., 2010). At present, the standard chemotherapy of TNBC includes different combinations of anthracyclines and taxane (Gadi and Davidson, 2017). MBC is a devastating disease with a median survival time of 3 years (Caswell-Jin et al., 2018). Most MBC patients will receive systemic therapy, including chemotherapy, endocrine therapy, molecular targeted therapy and certain promising treatment methods such as immunotherapy (Zhu et al., 2021) and antibody drug conjugate (Bardia et al., 2021). However, in the past decades, the improvement of overall survival rate was limited (Siegel et al., 2021).

Bevacizumab is a humanized monoclonal IgG antibody produced by DNA technology in Chinese hamster ovary cells (Ferrara et al., 2004; Gerber and Ferrara, 2005). The human part (93%) constitutes the antibody framework, and the murine part (7%) constitutes the complementary determining regions for binding with vascular endothelial growth factor (VEGF) (Braghiroli et al., 2013). Bevacizumab targets all VEGF-A subtypes, preventing the binding of VEGF-A with endothelial cell surface receptors, VEGF receptor (VEGFR)-1 (Flt-1) and VEGFR-2 (KDR/FLk-1) (Ferrara et al., 2004; Gerber and Ferrara, 2005). The inhibition of VEGF-A leads to the regression of tumor blood vessels and formation of new blood vessels, which results in inhibition of tumor growth. Bevacizumab could improve the delivery of other chemotherapy drugs by normalizing tumor blood vessels and reducing elevated interstitial pressure, which promotes the efficacy of suppressing the tumor growth and metastasis (Jain, 2001; Willett et al., 2004). It has been proved that in pre-clinical models, vascular degeneration occurs rapidly after starting the anti VEGF therapy. After the treatment, the formation and function of the surviving tumor vessels is temporarily “normalized”, resembling the normal vascular system. These changes reduce the intratumoral pressure and enhance the treatment efficacy of other anti-cancer therapies. Moreover, RCTs have showed improvements in tumor response rate and progression-free survival (PFS) when Bevacizumab was combined with different agents in treating HER-2 negative metastatic breast cancer and TNBC (Miller et al., 2007; Pivot et al., 2009; Robert et al., 2009).

Nevertheless, Bevacizumab treatment demonstrated increased frequency of various AEs, such as hypertension, proteinuria and asthenia. Certain serious AEs will lead to higher risks of catastrophic consequences (Miller et al., 2005; Miller et al., 2007; Miles et al., 2010), which might limit its clinical application. Therefore, It is imperative to analyze the occurrence of AEs during its application on the treatment of HER-2-negative MBC or TNBC treatment. We conducted a systematic review and meta-analysis including data from published clinical trials, and serious AEs of any grade and grade ≥3 were taken into consideration according to the National Cancer Institute Common Toxicity Criteria (NCI-CTC) (National Cancer Institute, 2022).

## 2 Materials and methods

### 2.1 Search strategy

**FIGURE 1:**
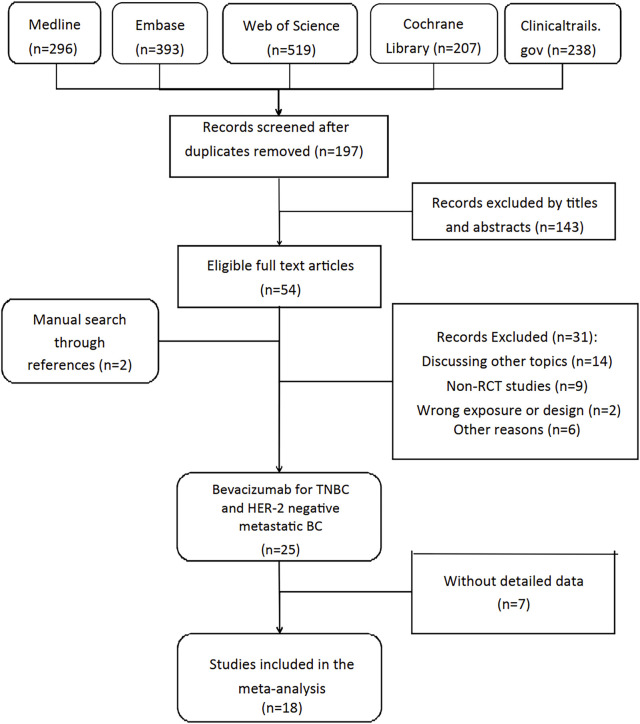
Flowchart of selection of included studies.

Our review followed the guidelines of Preferred Reporting Items for Systematic Reviews and Meta-Analyses (PRISMA) (Liberati et al., 2009), and it was registered in PROSPERO (registration number CRD42022354743) before literature search. Two reviewers (QGC and PH) searched Medline, Embase, Web of science and Cochrane databases updated to 1 Oct 2022 for RCTs independently. To expand the search range, we used the keywords “breast cancer”, “breast neoplasms”, “Bevacizumab”, “Avastin” and “adverse events”. The detailed search strategy used in MEDLINE database was available in supplementary material (see Figure 1). Clinicaltrials.gov was also searched for completed but unpublished RCTs with published results. Two researchers (SR and YZ) independently screened the titles and abstracts, articles meeting inclusion criteria were assessed for full-text review. Reference lists of eligible reviews and trials were searched for additional citations.

### 2.2 Inclusion criteria

Inclusion criteria were as followed: 1) phase III RCTs; 2) experiment group received treatment contained Bevacizumab, while control group received same treatment without Bevacizumab; 3) RCTs with available data of AEs. Besides, only RCTs published in English were included, and there was no restriction on age, sex, nationality, and race.

### 2.3 Data extraction

Two researchers (RL and YZ) independently extracted data from eligible articles and aggregated the results. The divergences were settled to consensus by consulting a third reviewer HYH. The information we extracted included: characteristic of study (author, year of publication, publication type, objective, type of disease, inclusion criteria, administration method, exposure and funding source), characteristic of patient (number of participates and age) and the outcomes. Outcomes were classified as primary outcomes and secondary outcomes. Primary outcomes included the sum of any grade AEs and grade ≥3 AEs in the experimental group and the control group. Secondary outcomes included the Specific incidence of various AEs in the experimental group and the control group. If the data was incomplete, the corresponding author would contact the author by email and invite them to send additional information for further research.

### 2.4 Quality assessment

Cochrane Risk of Bias Assessment Tool (CROBAT) was used by two researchers (XQX and QGC) to independently assess the quality of included studies. CROBAT included “Random sequence generation”, “Allocation concealment”, “Blinding of participants and personnel”, “Blinding of outcome assessment”, “Incomplete outcome data”, “Selective reporting”, and “Other bias” (see Supplementary Table S1). Each question had three answers: “Low risk”, “Moderate” and “High risk”. Researchers would assess the risk level of RCTs according to the published information. The decision was reached by consulting a third reviewer PH in the case of disagreements or failed consensus. Publication bias was evaluated by funnel plots and *p* ≤ 0.05 was considered statistically significant risk of bias. Small-study effects that led to potential reporting or publication bias could be calculated by Egger’s test. We used Grading of Recommendations, Assessment, Development, and Evaluation (GRADE) tool to evaluate the quality of evidence for each outcome. The GRADE tool classified evidence of outcomes into “High”, “Moderate”, “Low” and “Very low”. Each assessment could reduce or promote the level of quality. Specific rules were explained in Supplementary Table S2.

### 2.5 Statistical analysis

The HRs and 95% CIs for Disease-free survival (DFS) and Overall survival (OS) were collected, and they were weighted and combined by the generic inverse variance method (DerSimonian and Laird, 1986). Heterogeneity in the result of meta-analysis was assessed using Cochrane Q and I^2^ statistics with appropriate analysis models. When *p* ≤ 0.05 or I^2^ > 50%, the random effects model was used, and when *p* > 0.05 or I^2^ < 50%, the fixed effects model was used (Zintzaras and Ioannidis, 2005), and dichotomous data would be calculated by odds ratio (OR) with 95% CIs.

Subgroup analysis were carried out according to the dosage of Capecitabine, the number of cycles using Capecitabine, neoadjuvant or adjuvant chemotherapy, lymph node positivity or negativity, menopausal status. Sensitivity analysis was performed in the meta-analysis by excluding each study once at a time to check whether the effectiveness of outcome was determined by individual studies. All statistical analyses were performed using Review Manager 5.3 and STATA 16.0.

## 3 Results

### 3.1 Search result

Figure 1 demonstrated the detailed steps of the literature search, in which 1,653 studies were reviewed: 1,456 studies were excluded by screening titles and abstracts, the remaining 54 studies were reviewed in full text. After excluding 36 studies according to selection criteria, we included 18 RCTs articles with 12,664 female patients from 2010 to 2022.

### 3.2 Study characteristics

Table 1 displayed the characteristics of these articles. Among these RCTs, five RCTs focused on TNBC, while others discussed the HER-2 negative MBC. Moreover, we found 9 RCTs had the incidence of any grade AEs, and 8 RCTs with the incidence of grade ≥3 AEs. As for the detailed number of various AE cases, two researchers searched PubMed, Clinical Trial, Cochrane Library and further statistical analysis was performed. Based on the different types of included RCT, we conducted three subgroup analysis: the type of BC, the dosage of Bevacizumab, and the type of combined treatment (chemotherapy or endocrine therapy). We used Cochrane Risk of Bias Assessment Tool (CROBAT) to assess the quality of including studies, and Supplementary Table S1 demonstrated the risks of bias in our study that all RCTs are double-blinded and randomized. Table 1 showed that there no age limit for our inclusion, and there was no significant age difference between our experiment and control groups.

**TABLE 1:** The baseline characteristics.

Author year	Study ID	No.	Exposure	Comparator	Bevacizumab dose	Median age/y		Follow-up/m
						Case	Control	
*HER-2 (−)*								
David W Miles 2010	AVADO	736	Docetaxel + Bev* 7.5 mg/kg (n = 248)	Docetaxel + Placebo (n = 241)	7.5 mg/3w	53.9	53.5	13
David W Miles 2010	AVADO	736	Docetaxel + Bev* 15.0 mg/kg (n = 247)	Docetaxel + Placebo (n = 241)	15 mg/3w	53.6	53.5	13
David Miles 2017	MERiDiAN	481	Paclitaxel + Bev* (n = 239)	Paclitaxel + Placebo (n = 242)	10*2 mg/28 d	54.7	55.8	14.8
E Vrdoljak 2016	TANIA	494	Chemotherapy + Bev* (n = 247)	Chemotherapy (n = 247)	15 mg/3w or 10 mg/2w	54.7	55.8	32.1
Kathy D Miller 2005	AVF2119g	462	Capecitabine + Bev* (n = 229)	Capecitabine (n = 215)	15 mg/3w	52.0	51.0	17
Adam M Brufsky 2011	RIBBON-2	684	Chemotherapy + Bev* (n = 459)	Chemotherapy + Placebo (n = 225)	15 mg/3w or 10 mg/2w	55.6	55.0	15
Miguel Martin 2011	—	191	Paclitaxel + Bev* (n = 97)	Paclitaxel + Placebo (n = 94)	10*2 mg/3w	55.2	53.0	20
Nicholas J Robert 2011	RIBBON-1	615	Capecitabine + Bev* (n = 409)	Capecitabine + Placebo (n = 206)	15 mg/3w	56.0	57.0	15.6
Nicholas J Robert 2011	RIBBON-1	622	Tax/Anthra* + Bev* (n = 415)	Tax/Anthra* + Placebo (n = 207)	15 mg/3w	55.0	55.0	15.6
Norikazu Masuda 2017	MERiDiAN	54	Paclitaxel + Bev* (n = 24)	Paclitaxel + Placebo (n = 30)	15 mg/3w	52.5	59.5	14.8
Miguel Martín 2015	LEA	380	Endocrine Therapy + Bev* (n = 190)	Endocrine Therapy (n = 184)	15 mg/3w	64.0	66.0	21
Kathy Miller 2007	E2100	722	Paclitaxel + Bev* (n = 368)	Paclitaxel (n = 354)	10*2 mg/4w	56.0	55.0	30
Kathy D Miller 2018	E5130	2,986	Chemotherapy + Bev* (n = 1986)	Chemotherapy + Placebo (n = 1,000)	15 mg/3w	51.7	51.8	47
Maura N Dickler 2016	CALGB 40503	391	Endocrine Therapy + Bev* (n = 195)	Endocrine Therapy (n = 196)	15 mg/3w	55.5	58.9	39
*TNBC*								
R Bell 2017	BEATRICE	2,591	Chemotherapy + Bev* (n = 1,301)	Chemotherapy (n = 1,290)	5 mg/1w	—	—	56
Jonathan H Shepherd 2022	CALGB 40603	228	wP-ddAC* + Bev* (n = 113)	wP-ddAC* (n = 115)	10 mg/2w	—	—	189.6
Jonathan H Shepherd 2022	CALGB 40603	226	wPCarbo-ddAC* + Bev* (n = 113)	wPCarbo-ddAC* (n = 113)	10 mg/2w	—	—	189.6
V Diéras 2015	OAM486g	123	Onartuzumab + Paclitaxel + Bev* (n = 63)	Onartuzumab + Placebo + Paclitaxel (n = 60)	10*2 mg/28 d	53.0	54.5	24

*Bev: Bevacizumab; Carbo: carboplatin; ddAC: dose-dense doxorubicin and cyclophosphamide; wP: weekly paclitaxel.

### 3.3 Primary outcome

We analyzed 11 RCTs of any grade AEs and 9 RCTs of grade ≥3 AEs. As is shown in Table 2, the use of Bevacizumab was associated with increased incidence of any grade AEs (RR = 1.06, 95% CI 1.04–1.08, Rate = 64.55% vs. 70.59%). The application of Bevacizumab had a stronger correlation with the occurrence of grade ≥3 AEs (RR = 1.37, 95% CI 1.30–1.45, Rate = 52.59% vs. 41.32%). In general, the use of Bevacizumab is more closely related to grade ≥3 AEs. In addition, our data demonstrated that any grade AEs did not show significant statistical difference in the overall results and among the subgroups, and the level of evidence was not high. Besides, a higher incidence (RR = 1.37, *p* < 0.0001) of grade ≥3 AEs compared to the corresponding any grade AEs was demonstrated in Table 2. Therefore, we used grade ≥3 AEs group to illustrate in the following outcomes (similar results were shown in any grade AEs).

**TABLE 2:** Primary outcome.

	No. of RCTs	No. of patient	Rate (%)		RR (95% CI)	p-value*	GRADE*
			Case	Control			
Grade ≥ 3							
Total	9	5,943	52.59	41.32	1.37 [1.30, 1.45]	<0.0001*	Very low
Type of BC							
HER-2 negative	7	3,255	39.49	25.6	1.57 [1.41, 1.75]	<0.0001*	Low
TNBC	2	2,679	71.7	56.51	1.27 [1.20, 1.34]	<0.0001*	High
Administration							
>15 mg/3w	2	591	28.67	19.93	1.44 [1.07, 1.92]	0.01*	High
15 mg/3w	8	5,814	52.24	41.12	1.37 [1.30, 1.45]	<0.0001*	Low
ET or CT							
ET	2	765	31.17	13.42	2.32 [1.73, 3.12]	<0.0001*	Moderate
CT	8	5,591	52.97	43.25	1.33 [1.26, 1.40]	<0.0001*	High
Any grade							
Total	11	9,459	64.55	70.59	1.06 [1.04, 1.08]	<0.0001*	Very low
Type of BC							
HER-2 negative	9	6,780	53.42	57.37	1.10 [1.06, 1.14]	<0.0001*	Very low
TNBC	2	2,679	98.52	97.07	1.01 [1.00, 1.03]	0.01*	High
Administration							
>15 mg/3w	2	591	97.33	96.9	1.00 [0.98, 1.03]	0.77	Moderate
15 mg/3w	9	8,868	62.65	68.52	1.07 [1.05, 1.09]	<0.0001*	Very low
ET or CT							
ET	2	765	94.55	86.58	1.09 [1.04, 1.14]	0.0002*	Low
CT	9	8,694	62.28	68.91	1.06 [1.04, 1.08]	<0.0001*	Very low

*The low GRADE, is mostly due to the wide variety of adverse events included, leading to a significant increase in the heterogeneity of results.

*p-value ≤ 0.05.

Of all graded ≥3 AEs, proteinuria (RR = 9.22, 95% CI 4.49–18.93, Rate = 4.22% vs. 0.38%), mucosal inflammation (RR = 8.12, 95% CI 2.46–26.77, Rate = 3.49% vs. 0.43%), palmar-plantar erythrodysaesthenia syndrome (RR = 6.95, 95% CI 2.47–19.57, Rate = 6.01% vs. 0.87%), increased ALT (RR = 6.95, 95% CI 1.59–30.38, Rate = 3.13% vs. 0.24%) and hypertension (RR = 4.94, 95% CI 3.84–6.35, Rate = 9.44% vs. 2.02%) had the top five risk ratios. Only oedema peripheral (RR = 0.31, 95% CI 0.10–0.95, Rate = 0.8% vs. 2.6%) had a RR lower than 1 **(** Table 3).

**TABLE 3:** Total outcome.

	No. of RCTs	No. of patient	RR (95% CI)*	Rate (%)*		p-value*	GRADE
				Case	Control		
***Grade* ≥ *3***							
Proteinuria	7	3,639	9.22 [4.49, 18.93]	4.22	0.38	<0.0001*	Moderate
mucosal inflammation	3	1,444	8.12 [2.46, 26.77]	3.49	0.43	0.0006*	High
palmar-plantar erythrodysaesthenia syndrome	2	961	6.95 [2.47, 19.57]	6.01	0.87	0.0002*	High
ALT increased	3	830	6.95 [1.59, 30.38]	3.13	0.24	0.01*	High
Hypertension	14	8,507	4.94 [3.84, 6.35]	9.44	2.02	<0.0001*	Low
AST increased	5	2,430	3.04 [1.37, 6.70]	2.03	0.67	0.006*	High
Stomatitis	6	5,195	2.65 [1.62, 4.33]	2.54	0.82	0.0001*	High
Bleeding	7	3,779	2.31 [1.05, 5.11]	1.34	0.52	0.04*	High
Infection	7	5,329	2.14 [1.39, 3.30]	2.34	1.08	0.0006*	High
center ventricular dysfunction	6	5,847	2.14 [1.31, 3.52]	2.04	0.85	0.003*	High
Headache	5	4,863	2.13 [1.41, 3.21]	3.31	1.34	0.0003*	High
Vomiting	11	6,994	1.49 [1.08, 2.04]	2.26	1.36	0.01*	High
Leukopenia	8	2,938	1.48 [1.08, 2.02]	6.09	4.16	0.01*	High
Fatigue	11	6,994	1.47 [1.21, 1.77]	8.02	5.09	<0.0001*	High
Diarrhea	10	6,331	1.36 [1.05, 1.77]	3.97	3.24	0.02*	Moderate
febrile neutropenia	9	8,644	1.34 [1.12, 1.60]	6.16	5.12	0.001*	High
Neutropenia	11	9,270	1.34 [1.18, 1.54]	8.06	7.1	<0.0001*	High
peripheral sensory neuropathy	11	7,574	1.19 [1.01, 1.40]	7.73	6.77	0.04*	High
oedema peripheral	2	961	0.31 [0.10, 0.95]	0.8	2.6	0.04*	High
***Any grade***							
Proteinuria	9	8,144	3.90 [3.29, 4.63]	12.79	3.98	<0.0001*	Low
Epistaxis	7	4,971	3.63 [3.19, 4.13]	35.34	9.51	<0.0001*	Low
Dysphonia	4	3,977	3.59 [2.60, 4.97]	8.34	2.27	<0.0001*	High
Hypertension	11	8,800	3.22 [2.88, 3.59]	24.34	8.7	<0.0001*	Low
Thrombocytopenia	4	1,692	2.76 [1.73, 4.42]	7.1	2.52	<0.0001*	High
neuropathy peripheral	3	4,653	2.72 [2.25, 3.30]	20.71	10.21	<0.0001*	Low
Bleeding	3	967	1.88 [1.54, 2.30]	38.57	20.96	<0.0001*	High
AST increased	4	3,795	1.55 [1.29, 1.86]	11.51	7.38	<0.0001*	High
mucosal inflammation	3	1,430	1.54 [1.25, 1.90]	24.01	15.03	<0.0001*	High
center ventricular failure	4	6,029	1.54 [1.32, 1.81]	11.43	8.43	<0.0001*	High
lacrimation increased	3	3,506	1.50 [1.30, 1.73]	21.65	13.78	<0.0001*	High
Stomatitis	7	4,980	1.50 [1.39, 1.63]	39.7	26.46	<0.0001*	Moderate
Headache	8	7,826	1.48 [1.34, 1.62]	19.65	16.04	<0.0001*	High
Infection	2	629	1.46 [1.14, 1.87]	30.46	20.72	0.002*	Moderate
ALT increased	4	3,795	1.40 [1.16, 1.68]	10.47	7.43	0.0004*	High
musculoskeletal pain	4	3,977	1.33 [1.10, 1.59]	11.97	8.98	0.003*	High
Cough	5	4,368	1.32 [1.15, 1.52]	17.41	12.93	<0.0001*	High
palmar-plantar erythrodysaesthenia syndrome	4	3,989	1.30 [1.11, 1.51]	15.35	11.43	0.0009*	High
Pyrexia	5	4,460	1.24 [1.08, 1.42]	16.9	13.47	0.003*	High
decreased appetite	5	4,460	1.22 [1.08, 1.37]	21.85	17.69	0.001*	High
Diarrhea	10	5,974	1.19 [1.11, 1.29]	32.3	26.53	<0.0001*	High
hand-foot syndrome	2	927	1.18 [1.03, 1.35]	46.84	39.51	0.02*	High
Constipation	7	5,024	1.12 [1.03, 1.23]	28.19	25.11	0.01*	High
Neutropenia	9	7,900	1.12 [1.03, 1.20]	25.84	24.83	0.004*	Moderate
oedema peripheral	5	4,097	0.76 [0.68, 0.87]	16.47	20.94	<0.0001*	Moderate
Oedema	2	947	0.44 [0.29, 0.65]	6.43	14.75	<0.0001*	High
Hypoesthesia	2	947	0.31 [0.14, 0.68]	1.56	5.07	0.004*	High

*The above results are sorted according to the RR, results.

*Results with RR ≥ 5 have been highlighted and bolded. Results with 5 > RR ≥ 2 and RR ≤ 1have only been bolded.

*p-value ≤ 0.05.

*coloring if rate >5% in Grade ≥3 AEs, and rate >20% in Any grade AEs.

### 3.4 Secondary outcome

We displayed the results of each subgroup in Tables 4–6. Table 4 demonstrated that the AEs of Bevacizumab are more obvious in HER-2 negative breast cancer than in TNBC (RR_HER-2(−) MBC_ = 1.57 [1.41, 1.75], RR_TNBC_ = 1.27 [1.20, 1.34], 95% CI, *p* = 0.0007). Most of the RCTs administered 15 mg Bevacizumab every 3 weeks, and in Table 5 our study showed there was no significant difference in the occurrence of AEs between 15 mg/3w and a dose over 15 mg/3w (RR_15mg/3w_ = 1.37 [1.30, 1.45], RR_>15mg/3w_ = 1.44 [1.07, 1.92], 95% CI, *p* = 0.75). Additionally, Table 6 showed in the subgroup treated with ET, the incidence of grade ≥3 AE was obviously higher than that treated with chemotherapy (CT) (RR_ET_ = 2.32 [1.73, 3.12], RR_CT_ = 1.33 [1.26, 1.40], 95%CI, *p* = 0.0003). The following subgroup analysis is all about specific AE data.

**TABLE 4:** Subgroup outcome of Type of BC.

	No. of RCTs	No. of patient	RR (95% CI)*	Rate (%)*		p-Value	GRADE
				Case	Control		
***Grade* ≥ *3***							
***TNBC***							
Hypertension	2	454	9.11 [2.49, 33.24]	9.73	0.88	0.0008*	High
febrile neutropenia	3	3,013	1.49 [1.13, 1.96]	7.73	5.20	0.005*	High
Neutropenia	3	3,013	1.40 [1.15, 1.71]	10.77	7.74	0.001*	High
***HER-2*** (***−***)							
proteinuria	7	3,639	9.22 [4.49, 18.93]	4.22	0.38	<0.0001*	Moderate
mucosal inflammation	3	1,444	8.12 [2.46, 26.77]	3.49	0.43	0.0006*	High
palmar-plantar erythrodysaesthenia syndrome	3	1,430	6.95 [2.47, 19.57]	6.01	0.87	0.0002*	High
hypertension	12	8,053	4.80 [3.72, 6.21]	13.87	2.10	<0.0001*	Moderate
fatigue	9	6,470	1.47 [1.20, 1.80]	7.65	4.71	0.0002*	High
neutropenia	8	6,707	1.31 [1.10, 1.56]	7.08	6.72	0.003*	High
peripheral sensory neuropathy	9	7,120	1.19 [1.01, 1.41]	13.02	7.01	0.04*	High
oedema peripheral	2	961	0.31 [0.10, 0.95]	0.80	2.60	0.04*	High
***Any grade***							
***TNBC***							
epistaxis	2	2,679	5.94 [4.78, 7.39]	37.63	6.32	<0.0001*	Moderate
headache	2	2,679	1.51 [1.33, 1.71]	33.85	22.50	<0.0001*	High
diarrhea	2	2,679	1.22 [1.09, 1.37]	33.41	27.31	0.0007*	Low
***HER-2*** (***−***)							
proteinuria	8	5,585	3.09 [2.56, 3.73]	11.88	5.16	<0.0001*	Low
Thrombocytopenia	4	1,692	2.76 [1.73, 4.42]	7.10	2.52	<0.0001*	High
neuropathy peripheral	2	3,365	2.72 [2.25, 3.30]	26.67	10.21	<0.0001*	Low
epistaxis	5	2,292	2.37 [2.01, 2.79]	32.75	13.35	<0.0001*	Moderate
hypertension	10	6,241	2.37 [2.09, 2.68]	20.43	10.46	<0.0001*	Low
urinary tract infection	4	1,423	2.01 [1.38, 2.93]	9.46	4.52	0.0003*	Moderate
bleeding	3	967	1.88 [1.54, 2.30]	38.57	20.96	<0.0001*	High
stomatitis	6	2,421	1.78 [1.53, 2.08]	27.73	14.98	<0.0001*	High
lacrimation increased	2	947	1.57 [1.32, 1.87]	45.61	29.03	<0.0001*	High
mucosal inflammation	3	1,430	1.54 [1.25, 1.90]	24.01	15.03	<0.0001*	High
infection	2	629	1.46 [1.14, 1.87]	30.46	20.72	0.002*	Moderate
ALT/AST increased	3	1,236	1.44 [1.16, 1.77]	21.86	12.23	0.0008*	High
cough	4	1809	1.33 [1.08, 1.65]	18.39	13.33	0.008*	High
palmar-plantar erythrodysaesthenia syndrome	3	1,430	1.30 [1.09, 1.55]	29.29	22.62	0.004*	High
arthralgia	4	4,232	1.24 [1.09, 1.41]	13.27	14.98	0.0008*	High
neutropenia	7	5,221	1.20 [1.07, 1.35]	20.41	16.65	0.002*	High
hand-foot syndrome	2	927	1.18 [1.03, 1.35]	46.84	39.51	0.02*	High
diarrhea	8	3,295	1.17 [1.06, 1.29]	31.42	25.87	0.002*	High
oedema peripheral	3	1,418	0.66 [0.55, 0.78]	21.97	32.98	<0.0001*	Moderate
oedema	2	947	0.44 [0.29, 0.65]	6.43	14.75	<0.0001*	High
hypoesthesia	2	947	0.31 [0.14, 0.68]	1.56	5.07	0.004*	High

*The above results are sorted according to the RR, results.

*Results with RR ≥ 5 have been highlighted and bolded. Results with 5 > RR ≥ 2 and RR ≤ 1have only been bolded.

*p-value ≤0.05.

*coloring if rate >5% in Grade ≥3 AEs, and rate >20% in Any grade AEs.

**TABLE 5:** Subgroup outcome of Administration.

	No. of RCTs	No. of patient	RR (95% CI)*	Rate (%)*		p-value*	GRADE
				Case	Control		
***Grade* ≥ *3***							
***>15 mg/3w***							
nausea	2	656	6.70 [0.83, 53.89]	1.80	0.00	0.07	High
diarrhea	2	656	5.42 [0.96, 30.66]	2.40	0.31	0.06	High
hypertension	2	656	3.20 [1.61, 6.39]	9.59	3.11	0.001*	High
peripheral sensory neutropenia	2	656	1.90 [1.05, 3.45]	8.98	4.66	0.03*	High
***15 mg/3w***							
proteinuria	7	3,639	9.22 [4.49, 18.93]	4.22	0.38	<0.0001*	High
mucosal inflammation	3	1,444	8.12 [2.46, 26.77]	3.49	0.43	0.0006*	High
palmar-plantar erythrodysaesthenia syndrome	2	961	6.95 [2.47, 19.57]	6.01	0.87	0.0002*	High
ALT increased	3	828	6.92 [1.58, 30.25]	3.13	0.24	0.01*	High
hypertension	12	7,851	5.20 [3.97, 6.81]	9.41	1.91	<0.0001*	High
vomiting	9	6,268	1.62 [1.10, 2.39]	0.23	2.89	0.02*	High
fatigue	9	6,268	1.51 [1.24, 1.84]	8.46	5.26	<0.0001*	High
leukopenia	7	2,467	1.49 [1.07, 2.05]	6.69	4.54	0.02*	High
febrile neutropenia	9	8,644	1.34 [1.12, 1.60]	6.16	4.95	0.001*	High
neutropenia	10	9,249	1.28 [1.12, 1.47]	7.55	6.98	0.0004*	High
***Any grade***							
***>15 mg/3w***							
hypertension	2	656	2.51 [1.83, 3.44]	33.53	12.95	<0.0001*	High
epistaxis	2	591	2.16 [1.65, 2.83]	42.33	19.59	<0.0001*	High
neutropenia	2	591	1.51 [1.14, 2.00]	31.00	20.62	0.004*	High
diarrhea	3	776	1.34 [1.11, 1.62]	41.67	31.05	0.003*	High
***15 mg/3w***							
epistaxis	4	3,911	4.80 [4.03, 5.72]	32.58	6.62	<0.0001*	High
proteinuria	8	7,675	4.07 [3.41, 4.85]	13.26	4.00	<0.0001*	High
hypertension	8	7,675	3.59 [3.17, 4.06]	24.03	7.86	<0.0001*	High
Thrombocytopenia	4	1,692	2.76 [1.73, 4.42]	7.10	2.52	<0.0001*	High
headache	5	6,766	1.53 [1.38, 1.70]	18.49	15.05	<0.0001*	High
mucosal inflammation	2	961	1.52 [1.13, 2.05]	18.77	11.65	0.005*	High
stomatitis	4	3,855	1.44 [1.32, 1.56]	41.36	28.93	<0.0001*	High
pyrexia	3	3,520	1.26 [1.07, 1.48]	16.22	12.75	0.005*	High
decreased appetite	3	3,520	1.22 [1.06, 1.40]	20.40	16.51	0.005*	High
diarrhea	6	4,729	1.17 [1.07, 1.28]	28.20	23.70	0.0009*	High
neutropenia	6	6,840	1.09 [1.01, 1.18]	33.48	25.59	0.03*	High
fatigue	6	7,175	1.09 [1.01, 1.18]	24.29	25.99	0.02*	High
oedema peripheral	2	3,037	0.76 [0.64, 0.90]	13.30	17.00	0.001*	High

*The above results are sorted according to the RR, results.

*Results with RR ≥ 5 have been highlighted and bolded. Results with 5 > RR ≥ 2 and RR ≤ 1have only been bolded.

*p-value ≤0.05.

*coloring if rate >5% in Grade ≥3 AEs, and rate >20% in Any grade AEs.

**TABLE 6:** Subgroup outcome of ET or CT.

	No. of RCTs	No. of patient	RR (95%CI)*	Rate (%)*		p-value*	GRADE
				Case	Control		
***Grade* ≥ *3***							
***ET***							
proteinuria	2	717	8.66 [5.43, 13.79]	8.82	0.00	0.0006*	High
hypertension	2	717	2.48 [2.04, 3.02]	19.28	2.26	<0.0001*	High
AST increased	2	765	1.51 [1.21, 1.88]	2.60	0.53	0.04*	High
CT							
mucosal inflammation	3	1,444	8.12 [2.46, 26.77]	3.49	0.43	0.0006*	High
palmar-plantar erythrodysaesthenia syndrome	2	961	6.95 [2.47, 19.57]	6.01	0.87	0.0002*	High
proteinuria	5	2,922	6.18 [2.79, 13.67]	3.23	0.49	<0.0001*	High
hypertension	12	7,790	4.53 [3.45, 5.93]	8.88	1.99	<0.0001*	Moderate
Fatigue	9	6,159	1.44 [1.19, 1.74]	8.69	5.80	0.0002*	High
neutropenia	11	9,720	1.34 [1.18, 1.54]	8.06	10.38	<0.0001*	High
febrile neutropenia	9	8,644	1.34 [1.12, 1.60]	6.16	5.12	0.001*	High
***Any grade***							
***ET***							
hemorrhage	2	765	10.98 [3.73, 32.30]	10.13	0.79	<0.0001*	High
proteinuria	2	765	8.66 [5.43, 13.79]	40.78	4.74	<0.0001*	High
Fatigue	2	765	5.13 [3.59, 7.34]	31.17	16.84	<0.0001*	High
Diarrhea	2	765	3.22 [1.60, 6.50]	8.05	2.37	0.001*	High
hypertension	2	765	2.48 [2.04, 3.02]	60.00	24.21	<0.0001*	Low
Thrombocytopenia	2	765	2.44 [1.43, 4.18]	10.65	4.21	0.001*	High
AST increased	2	765	1.51 [1.21,1.88]	29.87	19.47	0.0002*	High
*** CT***							
dysphonia	4	3,977	3.59 [2.60, 4.97]	8.34	2.27	<0.0001*	High
Epistaxis	6	4,580	3.56 [3.12, 4.05]	37.25	10.25	<0.0001*	Low
hypertension	9	8,035	3.48 [3.05, 3.97]	21.32	7.01	<0.0001*	Low
proteinuria	7	7,379	3.24 [2.69, 3.90]	10.24	3.89	<0.0001*	Low
gingivitis	2	947	2.85 [1.31, 6.21]	5.26	1.84	0.008*	High
Bleeding	3	967	1.88 [1.54, 2.30]	38.57	20.96	<0.0001*	High
mucosal inflammation	3	1,430	1.54 [1.25, 1.90]	24.01	15.03	<0.0001*	High
lacrimation increased	3	3,506	1.50 [1.30, 1.73]	21.65	13.78	<0.0001*	High
stomatitis	6	4,606	1.49 [1.38, 1.61]	42.22	28.41	<0.0001*	High
headache	7	7,435	1.48 [1.34, 1.63]	18.56	15.21	<0.0001*	High
cough	4	3,977	1.32 [1.14, 1.52]	18.93	14.19	0.0001*	High
palmar-plantar erythrodysaesthenia syndrome	4	3,989	1.30 [1.11, 1.51]	15.35	11.43	0.0009*	High
diarrhea	8	5,209	1.28 [1.14, 1.44]	35.79	30.14	<0.0001*	High
pyrexia	5	4,460	1.24 [1.08, 1.42]	16.90	13.47	0.003*	High
decreased appetite	5	4,460	1.22 [1.08, 1.37]	21.85	17.69	0.001*	High
constipation	7	5,024	1.12 [1.03, 1.23]	28.19	25.11	0.01*	High
neutropenia	8	7,526	1.11 [1.03, 1.19]	26.50	25.90	0.009*	High
oedema peripheral	5	4,141	0.85 [0.75, 0.96]	16.47	20.94	0.01*	High
oedema	2	947	0.44 [0.29, 0.65]	6.43	14.75	<0.0001*	High

*The above results are sorted according to the RR, results.

*Results with RR ≥ 5 have been highlighted and bolded. Results with 5 > RR ≥ 2 and RR ≤ 1have only been bolded.

*p-value ≤ 0.05.

*coloring if rate >5% in Grade ≥3 AEs, and rate >20% in Any grade AEs.

#### 3.4.1 Subgroup analysis of breast cancer type

Table 5 demonstrated the AEs result of TNBC vs. HER-2 negative MBC. 1) TNBC: Among the grade ≥3 AEs of TNBC, hypertension (Rate: 9.73% vs. 0.88%, RR = 9.11), febrile neutropenia (Rate: 7.73% vs. 5.20%, RR = 1.49) and neutropenia (Rate: 10.77% vs. 7.74%, RR = 1.40) were notable. In any grade AEs, the differences of epistaxis (Rate: 37.63% vs. 6.32%, RR = 5.94), headache (Rate: 33.85% vs. 22.50%, RR = 1.51) and diarrhea (Rate: 33.41% vs. 27.31%, RR = 1.22) between TNBC group and HER-2 negative group are significant. 2) HER-2 negative metastatic breast cancer: as for the HER-2 negative MBC, in grade ≥3 AEs, hypertension was remarkable with the incidence of 13.87% in experimental group (Rate: 13.87% vs. 2.10%, RR = 4.80). In addition, proteinuria (Rate: 4.22% vs. 0.38%, RR = 9.22), mucosal inflammation (Rate: 3.49% vs. 0.43%, RR = 8.12), palmar-plantar erythrodysesthesia syndrome (Rate: 6.01% vs. 0.87%, RR = 6.95) and fatigue (Rate: 7.65% vs. 4.71%, RR = 1.47) were notable. Among any grade AEs, proteinuria (Rate: 11.88% vs. 5.16%, RR = 3.09), thrombocytopenia (Rate: 7.10% vs. 2.52%, RR = 2.76), neuropathy peripheral (Rate: 26.67% vs. 10.21%, RR = 2.72), epistaxis (Rate: 32.75% vs. 13.35%, RR = 2.37) and hypertension (Rate: 20.43% vs. 10.46%, RR = 2.37) were the most prominent. Besides, we also found a decrease in the incidence of oedema peripheral (Rate: 1.56% vs. 5.07%, RR = 0.31) in the experimental group. Further detailed data were listed in Table 4.

#### 3.4.2 Subgroup analysis of the dosage of bevacizumab

In Table 5, we listed the AEs results of 15 mg/3w vs. >15 mg/3w. 1) 15 mg/3w: We concluded that 15 mg/3w Bevacizumab increased the incidence of several grade ≥3 AEs significantly, such as proteinuria (Rate: 4.22% vs. 0.38%, RR = 9.22), mucosal inflammation (Rate: 3.49% vs. 0.43%, RR = 8.12), palmar-plantar erythrodysesthesia syndrome (Rate: 6.01% vs. 0.87%, RR = 6.95) and increased ALT (Rate: 3.13% vs. 0.24%, RR = 6.95). Besides, several any grade AEs were increased in 15 mg/3w Bevacizumab group significantly, such as epistaxis (Rate: 32.58% vs. 6.62%, RR = 4.80), proteinuria (Rate: 13.26% vs. 4.00%, RR = 4.07) and thrombocytopenia (Rate: 7.10% vs. 2.52%, RR = 2.76). 2)>15 mg/3w: usage of Bevacizumab>15 mg/3w resulted in a higher incidence in nausea (Rate: 1.80% vs. 0.00%, RR = 6.70), diarrhea (Rate: 2.40% vs. 0.31%, RR = 5.42) and hypertension (Rate: 9.59% vs. 3.11%, RR = 3.20) in the grade ≥3 AEs group. In any grade AEs group, apart from hypertension (Rate: 33.53% vs. 12.95%, RR = 2.51), the use of Bevacizumab>15 mg/3w increased the incidence of epistaxis (Rate: 42.33% vs. 19.59%, RR = 2.16), and that of neutropenia (Rate: 31.00% vs. 20.62%, RR = 1.51). Further detailed data were shown in Table 5.

#### 3.4.3 Subgroup analysis of CT vs. ET

Table 6 displayed the AEs results of CT vs. ET. 1) ET: in endocrine therapy, the increased incidence of proteinuria (Rate: 8.22% vs. 0.00%, RR = 8.66), hypertension (Rate: 19.28% vs. 2.26%, RR = 2.48) and elevated aspartate transaminase (AST) (Rate: 2.60% vs. 0.53%, RR = 1.51) was obvious in grade ≥3 AEs. The common AEs of any grade were hemorrhage (Rate: 10.13% vs. 0.79%, RR = 10.98), proteinuria (Rate: 40.78% vs. 4.74%, RR = 8.66), fatigue (Rate: 31.17% vs. 16.84%, RR = 5.13) and diarrhea (Rate: 8.05% vs. 2.37%, RR = 3.22). ***2) CT:*** in chemotherapy, the most pronounced grade ≥3 AEs were mucosal inflammation (Rate: 3.49% vs. 0.43%, RR = 8.12), palmar-plantar erythrodysesthesia syndrome (Rate: 6.01% vs. 0.87%, RR = 6.95), proteinuria (Rate: 3.23% vs. 0.49%, RR = 6.18), and hypertension (Rate: 8.88% vs. 1.99%, RR = 4.53), and of any grade AEs, an increase in incidence were found in dysphonia (Rate: 8.34% vs. 2.37%, RR = 3.59) and epistaxis (Rate: 37.25% vs. 10.25%, RR = 3.56). Further detailed data were shown in Table 6.

## 4 Discussion

**FIGURE 2:**
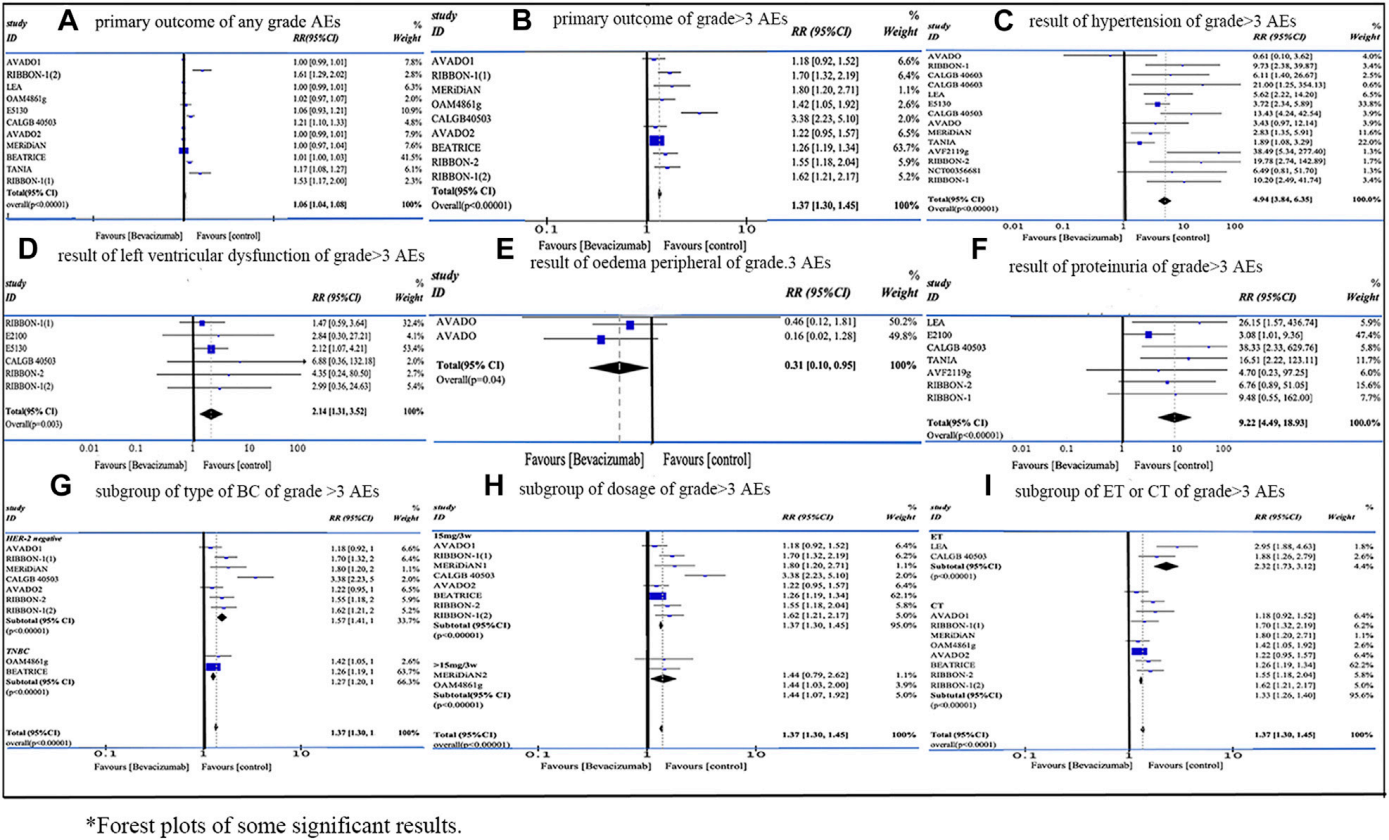
Forest plots of significant results.

Our meta-analysis included 18 RCTs with 12,664 female patients from Jan 2010 to Oct 2022. The application of Bevacizumab was associated with increased incidence of any grade AEs (RR = 1.06, 95% CI 1.04–1.08, Rate: 64.55% vs. 70.59%) and grade ≥3 AEs (RR = 1.37, 95% CI 1.30–1.45, Rate: 52.59% vs. 41.32%). As for the subgroup analysis of grade ≥3 AEs, 1) the AEs related to Bevacizumab were more obvious in HER-2 negative MBC than in TNBC (RR: 1.57 vs. 1.27); 2) there was no significant difference in the occurrence of AEs between 15 mg/3w and dosage over 15 mg/3w (RR: 1.37 vs. 1.44); 3) ET group demonstrated a higher incidence of grade ≥3 AEs than CT group (RR: 2.32 vs. 1.33). For grade ≥3 AEs, proteinuria (RR = 9.22, 95% CI 4.49–18.93, Rate: 4.22% vs. 0.38%), mucosal inflammation (RR = 8.12, 95% CI 2.46–26.77, Rate: 3.49% vs. 0.43%), palmar-plantar erythrodysaesthenia syndrome (RR = 6.95, 95% CI 2.47–19.57, Rate: 6.01% vs. 0.87%), elevated ALT (RR = 6.95, 95% CI 1.59–30.38, Rate: 3.13% vs. 0.24%), and hypertension (RR = 4.94, 95% CI 3.84–6.35, Rate: 9.44% vs. 2.02%) showed the top five risk ratios. Figure 2 shows the forest plot of some significant results.

Among all included types of AEs caused by Bevacizumab, the difference of hypertension was the most significant one. In our study, there was a significant difference in both any grade and grade ≥3 Hypertension (grade ≥3: RR = 4.94 95% CI 3.84–6.35, *p* < 0.00001; any grade: RR = 3.22 95% CI 2.88–3.59, *p* < 0.00001). For patients with HER-2 negative metastatic breast cancer, Miles DW et al. found a higher incidence in the high-dosage group (placebo 10.0%; 7.5 mg Bevacizumab 14.3%; 15 mg Bevacizumab 21.9%) (Miles et al., 2010). Similarly, another apparent difference was found in TNBC patients (placebo 5.1%; Bevacizumab 35.4%) by XX et al. (Bell et al., 2017). Although hypertension could be well managed with standard hypertension drugs and corresponding medications. However, if hypertension continues to deteriorate, it may lead to the discontinuation of Bevacizumab, and uncontrolled hypertension might incur left ventricular dysfunction. At the same time, the rising blood pressure would lead to a series of sequelae such as central nervous system (CNS) hemorrhage or hypertensive encephalopathy (Saif, 2009; Randall and Monk, 2010; Syrigos et al., 2011; European Medicines Agency, 2014). In recent years, a single nucleotide polymorphism of SV2C gene (Rs6453204) was discovered in the analysis of two breast cancer trials (E2100 (Miller et al., 2007) and E5103 (Miller et al., 2014)) by Schneider BP et al. They believed that hypertension caused by Bevacizumab could be predicted in advance by analyzing the genetic variation (Schneider et al., 2014). In conclusion, we recommended measuring blood pressure during the treatment. Bevacizumab combined with antihypertensive medications should be given to patients with HER-2 negative breast cancer and TNBC.

Our study demonstrated that proteinuria was associated with Bevacizumab and it could result in serious clinical consequences. Proteinuria accounted for a higher proportion in the experiment group in different grades of AEs in our results (grade ≥3: RR = 9.22 95% CI 4.49–18.93, *p* < 0.00001; any grade: RR = 3.90 95% CI 3.29–4.63, *p* < 0.00001). In previous RCTs, similar conclusions were drawn in both HER-2 negative MBC and TNBC (HER-2 negative MBC:23% vs. 13%; TNBC: 15.1% vs. 1.9%) (Vrdoljak et al., 2016; Bell et al., 2017). The study by Tanaka H et al. showed that proteinuria was a predictive factor in breast cancer patients receiving Bevacizumab treatment for improving the prognosis, since VEGF plays a role in maintaining the function of glomerular microvascular endothelial cells, and the inhibition of Bevacizumab would destroy the glomerular capillaries and result in the proteinuria (Saif and Mehra, 2006). Therefore, the occurrence of proteinuria indicates that Bevacizumab plays its pharmacological role in inhibiting VEGF. However, severe proteinuria might lead to the discontinuation of Bevacizumab treatment (Tanaka et al., 2018). The occurrence of proteinuria at all grades may be related to the dosage, and normally it does not cause serious consequences (European Medicines Agency, 2014). Thus, we recommended carrying out proteinuria detection before and during the treatment and making clinical intervention in time.

Bleeding is a possible AE caused by Bevacizumab, but serious bleeding events mainly occur in patients with non-small cell lung cancer. According to our results, an obvious difference occurred in both grade ≥3 AEs (RR = 2.31 95% CI 1.05–5.11, *p* = 0.04, Rate: 1.34% vs. 0.52%) and any grade AEs (RR = 1.88 95% CI 1.54–2.30, *p* < 0.00001, Rate: 38.57% vs. 20.96%). In the previous study, the risk of bleeding was dependent on the Bevacizumab dose and tumor type (Hang et al., 2011). Therefore, we strongly recommended discontinuing Bevacizumab application in patients with grade 3 or 4 bleeding. Moreover, we recommend that patients with central nervous system metastasis should be monitored for the signs and symptoms of bleeding discreetly, and Bevacizumab should be discontinued promptly in case of intracranial hemorrhage (Besse et al., 2010; European Medicines Agency, 2014).

In addition, cardiac dysfunction was one of the most serious AEs with low incidence. Several studies reported that Bevacizumab might result in a certain degree of cardiac toxicity, which may be induced and aggravated by anthracycline drugs. In addition, the use of Bevacizumab will lead to hypertension, and long-term hypertension will lead to left ventricular dysfunction (Rosa et al., 2016; Wittayanukorn et al., 2017). Similarly, in our results, there are a considerable number of patients suffering from decreased left ventricular function (grade ≥3: RR = 2.14 95% CI 1.31–3.52, *p* = 0.003, Rate: 2.04% vs. 0.85%). Bevacizumab recipients have an increased risk of arterial thrombotic events, especially in patients aged >65 years and those with diabetes or a medical history of arterial thromboembolism. Bevacizumab may lead to a series of diseases with serious consequences, such as stroke, transient ischemic attack and myocardial infarction (Scappaticci et al., 2007; Saif, 2009; Randall and Monk, 2010; European Medicines Agency, 2014). Therefore, special care should be given to elderly patients, especially those with a history of vascular disease.

Like Cardiac dysfunction, many AEs are serious but rare. 1) palmar-plantar erythrodysesthesia syndrome (grade ≥3: RR = 6.95 95% CI 2.47–19.57, *p* = 0.0002, Rate: 6.01% vs. 0.87%). 2) mucosal inflammation (grade ≥3: RR = 8.12 95% CI 2.46–26.77, *p* = 0.0006, 3.49% vs. 0.43%). 3) hand-foot syndrome (any grade: RR = 1.18 95% CI 1.03–1.35, *p* = 0.02, Rate: 46.84% vs. 39.51%). 4) thrombocytopenia (any grade: RR = 2.76 95% CI 1.73–4.22, *p* < 0.0001, Rate: 7.1% vs. 2.52%). Moreover, there were various AEs with low incidence and could be controlled and alleviated by timely treatment, including wound healing (Randall and Monk, 2010), gastrointestinal perforation (Saif, 2009), neutropenia (grade ≥3: RR = 1.34 95% CI 1.18–1.54, *p* < 0.0001, Rate: 8.06% vs. 7.1%), diarrhea (grade ≥3: RR = 1.36 95% CI 1.05–1.77, *p* = 0.02, Rate: 3.97% vs. 3.24%) etc. (see Table 3).

Bevacizumab was approved for the first time in the Europe (EU) in January 2005 (European Medicines Agency, 2006), and it was used as the first-line treatment for 1) metastatic colorectal cancer 2)advanced non-squamous non-small cell lung cancer (NSCLC) 3) metastatic breast cancer 4) advanced renal cell cancer 5) advanced epithelial ovarian cancer 6)fallopian tube cancer and 7) primary peritoneal cancer etc. (European Medicines Agency, 2014). In 2008, Bevacizumab was granted accelerated approval by US Food and Drug Administration (FDA) for patients with metastatic breast cancer. However, the clinical trials afterwards did not show significant improvement in OS, therefore the approval was withdrawn after comprehensive consideration of its improvement in OS and the possible AEs (Lenzer, 2011). To promote its clinical re-approval, there were various studies discussing the efficacy of Bevacizumab in patients with HER-2 negative metastatic breast cancer and TNBC. Miyashita M et al. conducted a meta-analysis in 2020 to assess the Risks and benefits of Bevacizumab combined with chemotherapy for advanced or metastatic breast cancer, and their results demonstrated the improvements of PFS [HR = 0.72, 95%CI 0.67–0.77, *p* < 0.00001]. However, the addition of Bevacizumab did not significantly improve the OS [HR = 0.95, 95% CI 0.87–1.03, *p* = 0.22], and the objective response rate (ORR) in the experimental treatment and chemotherapy-alone groups were 42% and 32% [HR = 1.74, 95% CI 1.26–1.71, *p* < 0.00001] (Miyashita et al., 2020). Similar conclusions were presented in several RCTs (Bell et al., 2017; von Minckwitz et al., 2014). For patients with HER2-negative locally recurrent or metastatic breast cancer, PFS was significantly longer in patients treated with Bevacizumab plus chemotherapy than in those with chemotherapy alone [HR = 0.75, 95% CI 0.61–0.93, *p* = 0.0068] in TANIA trial (von Minckwitz et al., 2014). For patients with TNBC, there was no statistically significant difference in OS between treatment arms and control arms [HR = 0.93, 95% CI0.74–1.17, *p* = 0.52), while 5-year invasive Disease-Free Survival (IDFS) rates were 77% (95% CI 75%–79%) in chemotherapy alone versus 80% (95% CI 77%–82%) in Bevacizumab group (Bell et al., 2017). These results were consistent with the reason that FDA rejected the approval of Bevacizumab for metastatic breast cancer treatment.

However, in almost all trials, the efficacy was statistically calculated with PFS as the primary endpoint. Therefore, there existed insufficient data to detect the change of OS as the improvement of OS was inconspicuous. In addition, the patients participating in RCTs usually receive additional treatment after stopping the assigned treatment, which may also affect the results of OS. What’s more, an opposite conclusion on OS had been demonstrated in the survey conducted by Delaloge et al., with a larger sample size and longer follow-up duration than previous RCTs. They assessed the efficacy of first-line paclitaxel with or without Bevacizumab, using the adjusted OS determined by matching the propensity score of several prognostic factors (Delaloge et al., 2016). The OS showed that the combination of paclitaxel and Bevacizumab group was significantly better than the paclitaxel group alone [HR = 0.67, 95% CI 0.60–0.75; median survival time: 27.7 vs. 19.8 months]. Combining the experimental results of studying the efficacy of Bevacizumab with our results for AEs, we believe that Bevacizumab is significant for the treatment of TNBC and HER-2 negative MBC. For possible AEs, most of them can be controlled by early prevention and timely interventions. In addition, our results can be used as a reference for clinicians.

Vascular endothelial growth factor is an effective angiogenesis regulator, which plays an important role in the pathogenesis of cancer. Various drugs targeting different sites of VEGF are available in clinical practice, such as Ramucirumab, a high affinity antibody targeting the extracellular domain of VEGFR-2, and Tyrosine kinase inhibitors (TKIs), an inhibitor of intracellular downstream signal transduction of VEGFRs, including sorafenib, cediranib, and sunitinib (Elgebaly et al., 2016; Mavratzas et al., 2019). However, these anti-VEGF drugs have shortcomings such as high price, poor efficacy, potentially life-threatening adverse reactions and drug resistance, which limit their clinical applications. In addition, we still lack methods to determine which patients will respond well to anti-angiogenesis therapy. Besides, although numerous studies had found potential predictive indicators to predict the efficacy of Bevacizumab, such as plasma levels of VEGF-A, VEGF-D, hepatocyte growth factor, interleukin-6/-8, inflammation-related markers, pentraxin-3 and ANG-2, their effectiveness remains to be further verified (Garcia et al., 2020). Therefore, future investigations are needed to further explore the ideal biomarkers to individualize the anti-VEGF treatment, reduce toxicity, and maximize the efficacy.

Our study has many advantages: 1) We only included patients with TNBC or HER-2 negative MBC. Their prognosis was less satisfactory with a higher risk of AEs, and the results were more targeted and clinically meaningful. 2) We divided the results into three subgroups for analysis, separately analyzed different AEs, and thoroughly analyzed those AEs with high incidence rate. 3) Our conclusions are more convincing since we included more updated RCT results, and our subgroup classification was more reasonable. However, there are still several limitations in our study. 1) Since the incidence rate of different AEs is quite different, the heterogeneity of the overall statistical results is high, but the heterogeneity of the results for individual AE is much lower. 2) Even if we try to contact the author by email, some of the included RCT data are incomplete, especially the data on clinicaltrail.gov. 3) Considering the length of the article, we only summarize preventive measures and treatment suggestions for major AEs.

## 5 Conclusion

The addition of Bevacizumab for TNBC and HER-2 negative MBC patients showed increased incidence of AEs especially for grade ≥3 AEs. The risk of developing different AEs varies mostly dependent on the type of breast cancer and the combined therapy. Moreover, much attention should be paid to proteinuria, mucosal inflammation, palmar-plantar erythrodysaesthenia syndrome, elevated ALT and hypertension.

## Data Availability

The original contributions presented in the study are included in the article/Supplementary Materials, further inquiries can be directed to the corresponding authors.
