# Supplementary material for: Adverse events of bevacizumab for triple negative breast cancer and HER-2 negative metastatic breast cancer: A meta-analysis
Source: Front Pharmacol. 2023 Jan 30;14:1108772. doi: 10.3389/fphar.2023.1108772 (PMC9922898; doi:10.3389/fphar.2023.1108772)
Supplement: Supplementary file 1 [file Table3.doc]

Table S3. Search Strategy of Medline

| Concept | Strategy | Result^#^ |
| --- | --- | --- |
| 1 | exp breast cancer / or breast neoplasms / | 334820 |
| 2 | (breast cancer * or breast neoplasms *).ti.ab | 10863 |
| 3 | 1 or 2 | 336599 |
| 4 | Bevacizumab *.ti,ab | 19404 |
| 5 | adverse events*.ti.ab | 191414 |
| 6 | 3 AND 4 AND 5 | 129 |
| 7 | (clinical trial or randomized controlled trial). pt. | 958831 |
| 8 | 6 AND 7 | 68 |

^#^updated to Jan 2^nd^ 2023

(((((breast cancer [MeSH Terms]) OR (breast neoplasms [MeSH Terms])) OR ((breast cancer Title/Abstract]) OR (breast neoplasms [Title/Abstract]))) AND ((Bevacizumab[Title/Abstract]))) AND ((adverse events[Title/Abstract]))) AND ((clinical trial[Publication Type]) OR (randomized controlled trial[Publication Type]))
