# Supplementary material for: Adverse events of bevacizumab for triple negative breast cancer and HER-2 negative metastatic breast cancer: A meta-analysis
Source: Front Pharmacol. 2023 Jan 30;14:1108772. doi: 10.3389/fphar.2023.1108772 (PMC9922898; doi:10.3389/fphar.2023.1108772)
Supplement: Supplementary file 2 [file Table1.doc]

Table S1. Outcome of CROBAT assessment

| Author Year | Trail Name | Random sequence generation | Allocation concealment | Blinding of participants and personnel | Blinding of outcome assessment | Incomplete outcome data | Selective reporting | Other bias |
| --- | --- | --- | --- | --- | --- | --- | --- | --- |
| 2010 Miles | NCT00333775（1） | Simple Random Sampling. | ﻿﻿docetaxel with  bevacizumab at a dose of 7.5 (bevacizumab7.5) or docetaxel with placebo | Blinded | Blinded | No | No | No |
| 2010 Miles | NCT00333775（2） | Simple Random Sampling. | docetaxel with  bevacizumab at a dose of 15 mg/kg (bevacizumab15)  or docetaxel with placebo | Blinded | Blinded | No | No | No |
| 2017 Miles | NCT01663727 | Simple Random Sampling. | ﻿intravenous paclitaxel 90 mg/m2 on  days 1, 8 and 15 with either placebo or bevacizumab  10 mg/kg intravenously | Blinded | Blinded | No | No | No |
| 2017 R Bell | NCT00528567 | Simple Random Sampling. | ﻿chemotherapy followed by observation or  combined with bevacizumab and followed by single-agent bevacizumab | Blinded | Blinded | No | No | No |
| 2016 E Vrdoljak | NCT01250379 | Simple Random Sampling. | ﻿standard second-line  chemotherapy either alone or in combination with bevacizumab 10 mg/kg every 2 weeks or  15 mg/kg every 3 weeks | Blinded | Blinded | No | No | No |
| 2005 D Miller | AVF2119g | Simple Random Sampling. | ﻿Patients randomized to the combination arm received bevacizumab (15 mg/kg) intravenously on day 1 of each 3-week cycle | Blinded | Blinded | No | No | No |
| 2011 M Brufsky | RIBBON-2 | Simple Random Sampling. | ﻿assigned 2:1 to either chemotherapy + bevacizumab or chemotherapy + placebo | Blinded | Blinded | No | No | No |
| 2011 Miguel Martin | NCT00356681 | Simple Random Sampling. | ﻿motesanib plus paclitaxel,  placebo plus paclitaxel, or open-label bevacizumab plus  paclitaxel. | Blinded | Blinded | No | No | No |
| 2011 J Robert | RIBBON-1(1) | Simple Random Sampling. | ﻿chemotherapy plus BV or chemotherapy plus  placebo | Blinded | Blinded | No | No | No |
| 2011 J Robert | RIBBON-1(2) | Simple Random Sampling. | ﻿chemotherapy plus BV or chemotherapy plus  placebo | Blinded | Blinded | No | No | No |
| 2022 H Shepherd | NCT00861705 | Simple Random Sampling. | ﻿Paclitaxel 80 mg/m2 /week × 12 /ddAC × 4  or  ﻿Paclitaxel 80 mg/m2 /week × 12 /ddAC × 4﻿  Bevacizumab 10 mg/kg / 2 weeks × 9 | Blinded | Blinded | No | No | No |
| 2022 H Shepherd | NCT00861705 | Simple Random Sampling. | Paclitaxel 80 mg/m2 /week × 12 /ddAC × 4  or  ﻿Paclitaxel 80 mg/m2 /week × 12 /ddAC × 4﻿  Bevacizumab 10 mg/kg / 2 weeks × 9 | Blinded | Blinded | No | No | No |
| 2017 Masuda | MERiDiAN | Simple Random Sampling. | ﻿paclitaxel 90 mg/m2 on Days 1, 8 and 15 with either placebo or bevacizumab 10 mg/kg on Days 1 and 15 | Blinded | Blinded | No | No | No |
| 2015 Martín | NCT00545077 | Simple Random Sampling. | ﻿ET alone(letrozole[2.5mgper  day] or fulvestrant [250 mg every 4 weeks], after an amendment) or ET-B  (bevacizumab 15 mg/kg body weight every 3 weeks) | Blinded | Blinded | No | No | No |
| 2015 V Diéras | NCT01186991 | Simple Random Sampling. | ﻿paclitaxel (90 mg/m2 days 1, 8, and 15), with onartuzumab (10 mg/kg days 1 and 15) or placebo, and bevacizumab (10 mg/kg  days 1 and 15) or placebo | Blinded | Blinded | No | No | No |
| 2007 Miller | NCT00028990 | Simple Random Sampling. | ﻿paclitaxel 90 mg/m2 IV infusion plus bevacizumab or paclitaxel 90 mg/m2 IV infusion | Blinded | Blinded | No | No | No |
| 2018 Miller | NCT00433511 | Simple Random Sampling. | ﻿placebo with doxorubicin and cyclophosphamide (AC)  followed by weekly paclitaxel (arm A), bevacizumab only during AC and paclitaxel (arm B) | Blinded | Blinded | No | No | No |
| 2016 N Dickler | NCT00601900 | Simple Random Sampling. | ﻿letrozole (2.5 mg orally per day) with or without bevacizumab (15 mg/kg  intravenously once every 3 weeks) | Blinded | Blinded | No | No | No |
